# Prophylactic Use of Pentoxifylline and Tocopherol for Prevention of Osteoradionecrosis of the Jaw after Dental Extraction in Post-Radiated Oral and Oropharyngeal Cancer Patients: An Initial Case Series

**DOI:** 10.3390/dj12040083

**Published:** 2024-03-22

**Authors:** Adepitan A. Owosho, Katherine A. DeColibus, Osariemen Okhuaihesuyi, Layne C. Levy

**Affiliations:** 1 Department of Diagnostic Sciences, College of Dentistry/Department of Otolaryngology—Head & Neck Surgery, College of Medicine, The University of Tennessee Health Sciences Center, 875 Union Avenue, Memphis, TN 38163, USA; 2 Department of Diagnostic Sciences, College of Dentistry, The University of Tennessee Health Sciences Center, Memphis, TN 38163, USA; 3 Missouri School of Dentistry and Oral Health, A.T. Still University, Kirksville, MO 63501, USA; 4 Advanced Education in General Dentistry, College of Dentistry, The University of Tennessee Health Sciences Center, Memphis, TN 38163, USA

**Keywords:** osteoradionecrosis, radiation therapy, radiotherapy, jaw, oral cancer, oropharyngeal cancer, pentoxifylline, vitamin E

## Abstract

Osteoradionecrosis of the jaw is a morbid complication of radiotherapy in patients with oral and oropharyngeal cancers that may be precipitated by dental extractions. Pentoxifylline and tocopherol (PENTO) has been utilized in the management of osteoradionecrosis and as prophylaxis for post-radiated head and neck oncology patients requiring an invasive dental procedure. This observational study aims to report the outcome of the prophylactic use of PENTO in the prevention of osteoradionecrosis of the jaw after dental extractions in post-radiated oral and oropharyngeal cancer patients and to review the current literature on this topic. Four post-radiated oral and oropharyngeal oncology patients were referred to the dental oncology clinic of the University Dental Practice, University of Tennessee Health Sciences Center for dental extractions. All four patients were prescribed pentoxifylline 400 mg BID (twice a day) and tocopherol 400 IU BID (oral tablets) for 2 weeks before extraction(s) and for 6 weeks after extraction(s). All patients were followed up every week after the second week post-extraction if feasible until the extraction site(s) healed (covered by mucosa). The assessment endpoint was defined as 6 weeks post-extraction with the outcomes assessed as using four categories determined by the area of exposed bone: complete healing (complete mucosal coverage of extraction site); partial healing (reduction in size of extraction site); no change; and progression (increase in size of the extraction site). At the assessment endpoint, all patients had complete healing of all extraction sites. The ORN rate at the patient level (0/4) and individual tooth level (0/8) was 0%. All patients tolerated the PENTO medications and no adverse effects from the use of these medications were reported. This limited study in addition to the other reviewed studies estimates the rate of ORN at the patient level as 3.2% (14/436) for post-radiated head and neck oncology patients after dental extractions/invasive oral procedures. In conclusion, this PENTO regimen can reduce/prevent the incidence of ORN in post-radiated head and neck oncology patients. This safe and cost-effective protocol (PENTO regimen) should be further evaluated as prophylaxis for post-radiated head and neck oncology patients requiring an invasive dental procedure. We recommend large prospective studies to be carried out to further validate these findings.

## 1. Introduction

In the year 2024, an estimated 58,450 individuals will be diagnosed with oral and pharyngeal cancer in the United States (U.S.) [1], a majority of which will be squamous cell carcinoma. Over 70% of these new cases occur in males and it is the eighth most common newly diagnosed malignancy in males [1]. The primary etiologies for these cancers are tobacco smoking and human papillomavirus (HPV), and the incidence of HPV-related oropharyngeal cancers is on the rise [2]. Approximately 70% of new oral and pharyngeal cancer cases are diagnosed at a late stage [3], thereby warranting the use of radiotherapy either as neoadjuvant or adjuvant therapy in combination with other therapeutic modalities such as surgery and chemotherapy. Oral and pharyngeal cancer patients typically receive radiation doses up to 60–70 Gy. Even with advanced radiation delivery techniques such as intensity-modulated radiation therapy (IMRT) and proton therapy, enabling improved tumor dose conformality and tissue/organ sparing capability, oral complications such as xerostomia, caries, trismus, and osteoradionecrosis (ORN) still exist [4,5,6,7,8].

**Figure 1:**
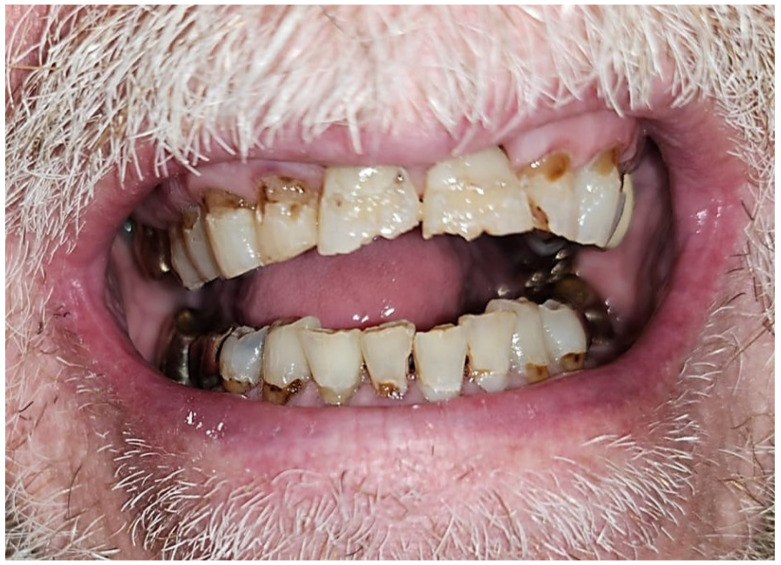
Post-radiated oral cancer survivor presenting to the dental oncology clinic with rampant dental caries.

Post-radiation oral and oropharyngeal cancer patients are prone to developing rampant dental caries secondary to xerostomia (Figure 1), inadequate quality/buffering action of saliva, and trismus which interferes with keeping good oral hygiene. This may result in non-restorable teeth, thereby requiring dental extractions. Tooth extraction/dentoalveolar surgery (trauma) are considered major risk factors for the development of ORN [9,10]. Other risk factors are radiation dose > 60 Gy, poor oral hygiene, tobacco smoking, and alcohol use [11,12,13]. ORN, a morbid complication of radiotherapy to the head and neck, is defined as an area of exposed necrotic bone in a previously irradiated area that does not heal over a period of 3–6 months [14]. Radiation to the jaw bone results in a decrease in vascularity resulting in hypoxia, hypovascularity, and hypocellularity [14]. These have been described as etiological factors in the development of ORN. The reported prevalence of ORN varies widely from 0.4 to 56% [15]. However, in the era of IMRT, 4.3–6.8% of patients who had radiation therapy using IMRT modality in the treatment of oral and oropharyngeal cancer developed ORN [11,12,13]. A recent study found the prevalence of ORN in head and neck cancer patients treated using proton therapy to be 10.6% (13/122) [7]. Although ORN is a rare disease, its devastating effect on a patient’s quality of life is profound. To date, there is no consensus on standard protocol for the management of ORN and emphasis has been made on its prevention. The management approaches employed have historically been wound care with chlorhexidine rinse; the use of antibiotic, pharmacologic management of pain; passive removal of exposed necrotic bone when indicated; hyperbaric oxygen therapy; and surgical treatment such as alveolectomy, maxillectomy, or segmental jaw resection and reconstruction with bone graft/flap in severe cases [16,17,18]. The prevention of ORN entails pre-radiation dental evaluation, prompt treatment of dental caries and periodontal disease, and dental extraction of grossly carious and periodontally involved teeth with poor prognosis. Invasive dental procedures should be completed not less than 2 weeks before the commencement of radiation therapy [19,20]. However, in real-world experiences, many post-radiation oral and pharyngeal cancer patients are faced with the need for extractions. The risk of ORN post-extraction in an irradiated oral/pharynx cavity is lifelong [21,22].

**Figure 2:**
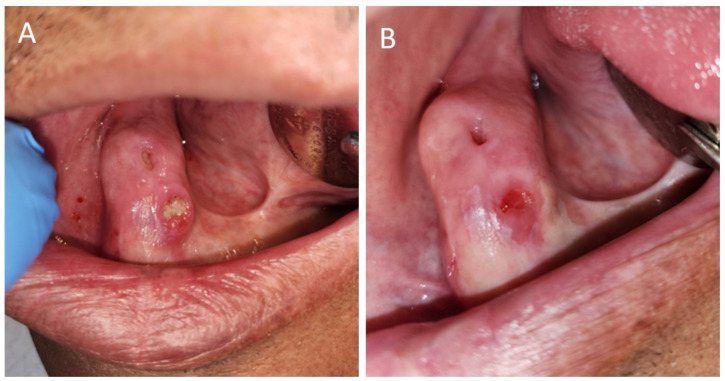
A 57-year-old male oral cancer survivor was referred to the dental oncology clinic for the management of osteoradionecrosis. (**A**) Osteoradionecrosis of the left alveolar ridge with exposed bone. (**B**) Area of exposed bone covered with mucosa after being on pentoxifylline and tocopherol for 3 months.

Pentoxifylline and tocopherol (PENTO) are now used in the management of ORN based on the new theory of radiation-induced fibrosis which states that the dysregulation of fibroblastic activity is the critical event in the pathogenesis of ORN [23]. A therapeutic regimen with PENTO (pentoxifylline 400 mg BID [twice daily] and tocopherol 400 IU BID) for managing ORN has been developed, showing noteworthy results in managing ORN (Figure 2). The study by Delanian et al. in 2011 reported the complete restoration and mucosal healing in all fifty-four refractory mandibular osteoradionecrosis cases by prolonged treatment with a pentoxifylline-tocopherol-clodronate combination in a phase II trial [24]. Subsequently, other studies have reported patients with complete mucosal coverage of exposed bone after PENTO treatment, ranging from 16.6% to 86.6% [25]. Pentoxifylline a methylxanthine derivative was originally approved by the U.S. Food and Drug Administration for the management of peripheral artery disease, such as intermittent claudication. Pentoxifylline improves peripheral blood flow by enhancing vasodilation, reduction in blood viscosity, and increasing erythrocyte flexibility. It also reduces inflammation and decreases fibrosis by inducing anti-tumor necrosis factor beta effects [26]. Tocopherol, a potent oxygen radical scavenger, reduces free radical damage during oxidative stress, thereby protecting cell membranes, reducing inflammation, and tissue fibrosis [26].

Given these findings, it has been hypothesized that the prophylactic use of PENTO in irradiated oral and pharyngeal cancer patients needing dental extractions may improve oral mucosa healing, therefore preventing ORN [27,28,29,30]. There are only five retrospective case studies published on the prophylactic use of PENTO to prevent ORN: four studies conducted in Europe, one study in India and none reported in the U.S. Across four retrospective case studies that utilized PENTO in a tablet form, the ORN rate in patients was 3.1% (12/387), and at the individual tooth level, it was 0.9% (11/1269) [31]. The fifth study used a liquid formulation of pentoxifylline and tocopherol and the majority of the patients (38/45) had only dental extractions carried out; the ORN rate in patients was 4.4% (2/45) [32]. The two ORN patients eventually healed after continual use of PENTO for 19 and 44 months [32].

This observational study aims to report the outcome of the prophylactic use of PENTO in the prevention of ORN of the jaw after dental extraction(s) in post-radiated oral and oropharyngeal cancer patients.

## 2. Patients and Methods

**Figure 3:**
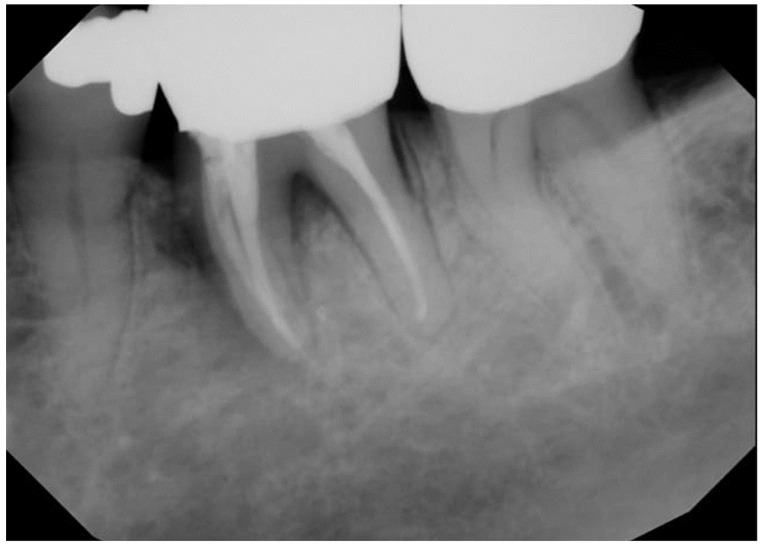
A 69-year-old male patient managed with 70 Gy radiation therapy for his base of tongue squamous cell carcinoma (Case 1) was referred to the dental oncology clinic on account of a painful tooth (#19) associated with a draining abscess. Patient was prescribed antibiotics for a week prior to dental extraction for the draining abscess.

**Figure 4:**
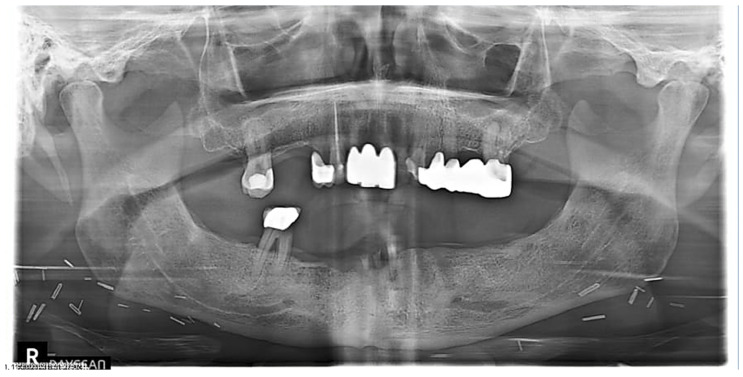
A 70-year-old male patient managed with 70 Gy radiation therapy for his right ventral tongue and floor of mouth squamous cell carcinoma (Case 2) was referred to the dental oncology clinic on account of painful teeth (#24, #25, #26, and #30) and oral rehabilitation.

This observational retrospective study was exempt from review by The University of Tennessee Health Sciences Center (UTHSC) Institutional Review Board. Head and neck oncology patients are routinely referred to the dental oncology clinic of the University Dental Practice, UTHSC for pre- and post-radiation therapy dental management. In the past 18 months, four post-radiated oral and oropharyngeal oncology patients who were referred for dental management required dental extraction(s) (Figure 3 and Figure 4). Patients were duly informed about the possible risk of developing ORN of the jaw after dental extractions and informed about the potential success of mitigating this risk with the use of PENTO (pentoxifylline 400 mg BID and tocopherol 400 IU BID) oral tablets administered prior to and continually after the extraction(s). The following medical conditions were ruled out: uncontrolled diabetes mellitus, prior history of ORN or MRONJ, history of stroke, ocular hemorrhage, kidney disease, liver disease, recent history of major surgery, peptic ulcer, and anti-thrombotic (anti-coagulant and anti-platelet) medication use. The following information was collected: Age and gender of the patient, tumor type, primary tumor site, radiation dose to primary tumor site, number of tooth/teeth extracted, and extracted tooth/teeth sites. In line with other studies [27,28,30], patients were categorized as high, moderate, or low risk for ORN after dental extraction, based on the proximity of the tooth to be extracted to the primary tumor/radiation field. High risk is defined as extraction(s) on the same side as the primary tumor and within the radiation-planned treatment volume. Moderate risk is defined as extraction(s) on the contralateral side of the primary tumor and/or opposite jaw location. Low risk is defined as extraction(s) in an area distant from the site of the primary tumor, for example, extraction of mandibular molar(s) in a patient with a laryngeal tumor. All patients were prescribed pentoxifylline 400 mg BID and tocopherol 400 IU BID for 2 weeks before extraction(s) and for 6 weeks after extraction(s). All patients signed informed consent and treatment waivers understanding that the risk for ORN remains even with the prophylactic use of PENTO. Only one patient was prescribed antibiotics for a week prior to dental extraction for the draining abscess, all other patients were not given antibiotics. All procedures were simple extractions using 2% lidocaine with epinephrine 1:100,000, elevators, and forceps. No mucosal flap was raised. Extraction site(s) were irrigated with chlorhexidine gluconate 0.12% oral rinse. All patients were followed up every week after the second week post-extraction if feasible until the extraction site(s) healed (covered by mucosa). The assessment endpoint was defined as 6 weeks post-extraction with the outcomes assessed using four categories determined by the area of exposed bone: complete healing (complete mucosal coverage of extraction site); partial healing (reduction in size of extraction site); no change; and progression (increase in size of the extraction site).

## 3. Results

**Figure 5:**
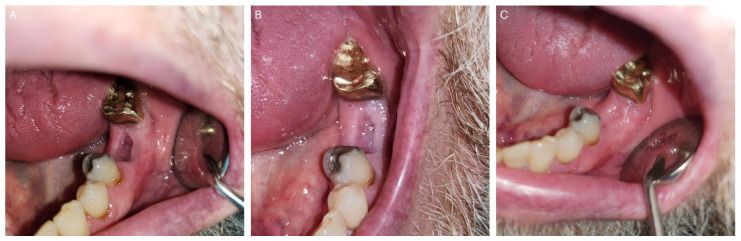
A 69-year-old male patient managed with 70 Gy radiation therapy for his base of tongue squamous cell carcinoma (Case 1) placed on pentoxifylline and tocopherol before extraction of tooth #19. (**A**) Clinical picture of the post-extraction site at 3 weeks. (**B**) Clinical picture of the post-extraction site at 5 weeks showing complete mucosa coverage of the extraction site. (**C**) Clinical picture of the post-extraction site at 23 weeks.

**Figure 6:**
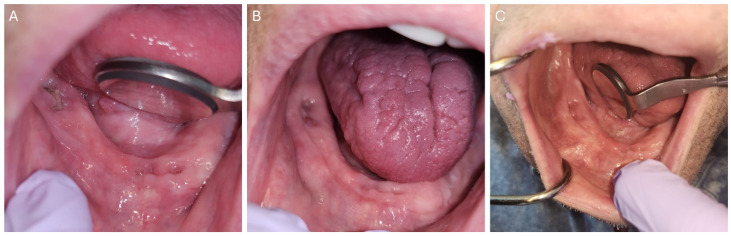
A 70-year-old male patient managed with 70 Gy radiation therapy for his right ventral tongue and floor of mouth squamous cell carcinoma (Case 2) placed on pentoxifylline and tocopherol before extraction of teeth #24, #25, #26, #30. (**A**) Clinical picture of post-extraction sites at 2 weeks. (**B**) Clinical picture of post-extraction sites at 5 weeks showing complete mucosa coverage of the extraction site. (**C**) Clinical picture of the post-extraction site at 7 weeks.

**Figure 7:**
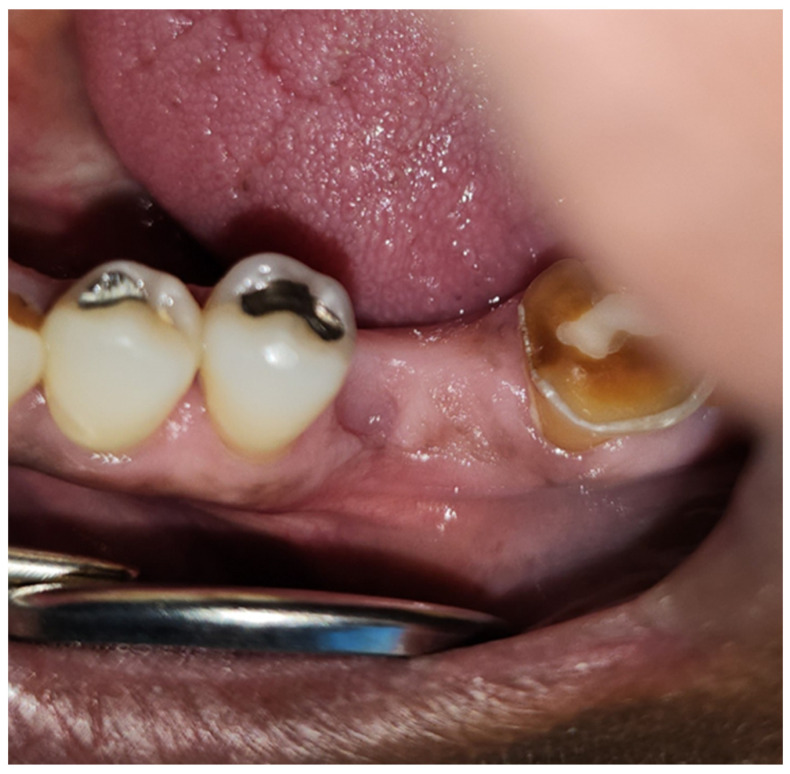
A 59-year-old female patient managed with 70 Gy radiation therapy for her right tonsil squamous cell carcinoma (Case 3) placed on pentoxifylline and tocopherol before extraction of tooth #19. A clinical picture of the post-extraction site at 4 weeks showing complete mucosa coverage of the extraction site.

Table 1 summarizes the clinical characteristics of the patients, tooth/teeth extracted, and treatment outcomes. There were four patients (male n = 3, female n = 1; ages 59–75 years). Three patients were managed for squamous cell carcinomas (Cases 1–3) and one patient for adenoid cystic carcinoma (Case 4). In two of the patients, the primary tumor location was in the oral cavity (ventral tongue/floor of mouth and palate/maxillary sinus) and in the other patients, the primary tumor location was in the oropharynx (base of tongue and tonsil). All patients received ≥ 66 Gy of radiation to the primary tumor site and were all treated with IMRT. Two patients had one tooth extracted, one patient had two teeth extracted, and one patient had four teeth extracted. Mandibular first molars were extracted in three patients, mandibular second molars were extracted in one patient, and mandibular incisors in one patient. Two patients were classified as having a high risk for ORN after dental extraction (Cases 1 and 2) and two were patients classified as having a moderate risk for ORN after dental extraction (Cases 3 and 4). At the assessment endpoint at 6 weeks post-extraction, all patients had complete healing of all extraction sites (Figure 5, Figure 6 and Figure 7). The ORN rate at the patient level (0/4) and individual tooth level (0/8) was 0%. All patients tolerated the PENTO medications and no adverse effects from the use of these medications were reported.

## 4. Discussion

ORN is the most morbid oral complication of radiation to the oral and oropharyngeal region. Dental extraction/dentoalveolar surgery (trauma) are considered major risk factors for the development of ORN [9,10]. The systematic review by Nabil and Samman places the incidence of ORN after dental extraction at the patient level as 7% and a more recent systematic review and meta-analysis by Balermpas et al. reported the risk of developing dental extraction-related ORN at the patient level as 7.6% in head and neck cancer patients treated with IMRT [33,34]. The reported prevalence of ORN in patients managed with proton therapy is 10.6% (13 out of 122) [7]. In these thirteen patients that developed ORN, the primary tumor site was in the oral cavity and oropharyngeal region, and there was no reported precipitating dental extraction, dentoalveolar surgery or trauma [7]. The two main historic prophylactic strategies used to prevent or decrease the risk of ORN after invasive dental treatment in a post-radiated head and neck cancer patient have been the use of antibiotics prophylaxis (AP) and hyperbaric oxygen therapy (HBOT) [35,36]. A randomized prospective clinical trial by Marx et al. comparing AP with prophylactic HBOT in the prevention of ORN reported the rates of ORN after dental extraction as 29.9% and 5.4%, respectively [36]. The randomized controlled trial by HOPON study comparing AP with prophylactic HBOT to prevent ORN reported the rates of ORN after dental extraction as 5.7% and 6.4%, respectively [37]. A systematic review by Nabil and Samman estimated the ORN rates after dental extraction using AP or prophylactic HBOT as 6% and 4% of patients, respectively, and 1.7% and 2.9% of individual teeth, respectively [33]. The systematic review by Fritz et al. on using prophylactic HBOT to prevent ORN reported the rate of ORN after dental extraction as 4.1% [38].

Based on the new theory of radiation-induced fibrosis where the dysregulation of fibroblastic activity is the critical event in the pathogenesis of ORN, PENTO is now utilized in the management of ORN. It has been proposed that the prophylactic use of PENTO in irradiated head and neck cancer patients needing dental extractions may improve oral mucosa healing, therefore preventing ORN [22,27,39,40,41,42,43,44,45,46,47]. A recent systematic review of four studies (Patel et al., Aggarwal et al., Samani et al., and Lombardi et al.) that prophylactically used PENTO prior to dental extractions in order to prevent ORN estimated the ORN rate at patient level as 3.1% and at individual tooth level as 0.9% [31]. For the fifth study (liquid formulation of PENTO), where the majority of the patients (38/45) had only dental extractions carried out (one patient had both extractions and implant placed and the remaining six patients had implants placed), the ORN rate at patient level was 4.4% (2/45) [32]. Both patients eventually healed after continual use of PENTO for 19 and 44 months. Table 2 summarizes the literature review on studies with prophylactic pentoxifylline and tocopherol use before dental extractions to prevent ORN. Comparing the use of PENTO to AP or prophylactic HBOT for the prevention of ORN after dental extraction clearly shows that PENTO has better results than AP or prophylactic HBOT in reducing the incidence of ORN after dental extraction in irradiated head and neck cancer patients. In this limited study of four patients, the ORN rates at the patient level and individual tooth levels were 0%.

At this time, the utility of prophylactic HBOT in the prevention of ORN after dental extraction cannot be justified since studies have now shown that at the least, prophylactic HBOT does not provide better outcomes when compared to PENTO and for the following reasons: lack of general and ready availability, logistical challenges, and cost. A total of 30 dives (20 pre-extraction and 10 post-extraction) in a compression chamber at 2.4 atmospheres, each for 90 min, totaling 45 h is recommended for a dental extraction. HBOT cost per dive ranges between USD 250 and 600, with amounts between USD 7500 and 18,000 for the 30 dives recommended for a dental extraction. In contrast, the medications used for the PENTO regimen are readily available in most pharmacies. Pentoxifylline is dispensed by prescription and tocopherol can be purchased over the counter. These medications can be used by patients without any special accommodations and are incomparably cheaper when compared with HBOT. An 8-week regimen of pentoxifylline and tocopherol costs between USD 44 and 112 and USD 22, respectively, making a total ranging between USD 66 and 134 for both medications. That is more than a 100-fold difference in cost between both options; this also holds true in the United Kingdom [29]. The major setbacks with pentoxifylline use are that it is contraindicated in patients with a history of a recent stroke, ocular hemorrhage, kidney problems, liver problems, recent major surgery, stomach ulcer, and patients on anti-thrombotic (anti-coagulant and anti-platelet) medication use. Also, the adverse effects of these medications such as nausea, vomiting, gastric irritation, headaches, vertigo, hot flushes, and epigastralgia may result in non-compliance with the medication use. A study carried out by Patel et al. found that 7.3% of patients could not tolerate these medications [27]. Aggarwal et al. reported in their study that 9.1% of patients developed adverse effects from the medications [28] and a study by Samani et al. reported that 4.8% of their patients developed well-recognized adverse effects to the medications [29]. In our limited study, no patient reported any adverse effects to the medications. This may be because our patients were on the medications for a shorter duration (8 weeks) than those in the other studies. However, no logical conclusion can be made due to the small sample size of our study. The use of the liquid formulation for both pentoxifylline and tocopherol by Patel et al. was to encourage patients who suffer from dysphagia to have an alternative option in using these medications [32]. Amongst all five studies, the duration of use of these medications varies from 1 week to 1 month preoperatively and 6 weeks to 3 months postoperatively (Table 2). A standardized duration may be required.

The use of photobiomodulation therapy (PBMT) has been encouraged for the prevention of ORN in post-radiated head and neck cancer patients requiring dental extraction [48]. The study by da Silva et al. used PBMT in conjunction with antibiotic therapy and surgical alveolotomy to ensure primary closure of the extraction sites [48]. Nineteen patients were enrolled in the test group and the extraction sites were exposed to PBMT using Flash Laser III (DMC, São Paulo, Brazil) at 40 mW, 100 J/cm^2^, 70 s/point, and 2.8 J/point on five points (total of 70 J/alveolus). The PBMT was applied four times every week [48]. All 19 patients had complete mucosal healing by 28 days post-extraction [48]. This is the only study on the use of PBMT prophylactically in the prevention of ORN in post-radiated head and neck cancer patients requiring dental extraction. More studies are encouraged to know its true efficacy. Also, the use of antibiotics can be a confounding factor in the results.

The PENTO protocol should be a game changer in oncology clinical practice. As stated earlier, the emphasis has been on the prevention of ORN. In some oncology clinical practices, patients are encouraged to extract all their teeth with the slightest pathoses, before beginning head and neck radiation therapy. A recent study by Buurman et al. estimated that 74% (1274/1759) of teeth extracted before head and neck radiation therapy have been removed redundantly based on a dosimetry dose mean of <40 Gy [49]. If the use of prophylactic PENTO, which is cheap and safe, can prevent/reduce ORN in irradiated patients, clinicians may be more comfortable preserving patient’s dentition to a reasonable limit. Studies have shown that loss of dentition in these patients affects their functional units, mastication, swallowing, and nutritional status, contributing to a decreased quality of life [50,51,52,53,54]. Many oral care providers are not comfortable carrying out invasive dental procedures such as root planning and extractions in post-irradiated oncology patients. As such, many head and neck cancer survivors must endure complications such as dentoalveolar infections, cellulitis, osteomyelitis, sepsis, and odontogenic cysts. These complications can be easily prevented by a simple dental extraction of a non-restorable tooth. Instituting PENTO as a standard protocol in the management of non-fractured ORN and as prophylaxis for post-radiated head and neck oncology patients requiring an invasive dental procedure may help alleviate these complications.

## 5. Conclusions

This limited study, in addition to the other reviewed studies, estimates the rate of ORN using PENTO protocol at the patient level as 3.2% (14/436) for post-radiated head and neck oncology patients after dental extractions/invasive oral procedures. The PENTO regimen can reduce/prevent the incidence of ORN in post-radiated head and neck oncology patients. This safe and cost-effective protocol (PENTO regimen) should be instituted as prophylaxis for post-radiated head and neck oncology patients requiring an invasive dental procedure. We recommend large prospective studies to be carried out to further validate these findings.

## Figures and Tables

**Table 1:** Clinical characteristics of the patients, tooth extracted, and outcome.

Case No	Gender	Age	Tumor Type	Tumor Site [Laterality]	Radiation Dose to Primary Tumor/Chemotherapy (Yes/No)	Extracted Teeth	Risk for ORN	Outcome
1	M	69	Squamous cell carcinoma	Base of Tongue (Oropharynx)	70 Gy/(Yes)	Mandibular First Molar (#19)	High	Complete Healing
2	M	70	Squamous cell carcinoma	Ventral Tongue/Floor of Mouth (Oral Cavity) [Right]	70 Gy/(Yes)	Mandibular Incisors and First Molar (#24, #25, #26, and #30)	High	Complete Healing
3	F	59	Squamous cell carcinoma	Tonsil (Oropharynx) [Right]	70 Gy/(Yes)	Mandibular First Molar (#19)	Moderate	Complete Healing
4	M	75	Adenoid cystic carcinoma	Hard palate/Maxillary Sinus (Oral Cavity) [Left]	66 Gy/(No)	Mandibular Second Molars (#18 and #31)	Moderate	Complete Healing

**Table 2:** Literature review on studies with prophylactic pentoxifylline and tocopherol use before dental extractions to prevent ORN.

Study	PENTO Regimen	Total Number of Patients Who Had Extractions (% of Oral and Oropharyngeal Tumor Sites)	Total Number of Teeth Extracted	ORN Rate by Patient	ORN Rate by Teeth
Patel et al. (2016) [27]	Pentoxifylline 400 mg BID and Vitamin E 1000 IU daily, 1 month preoperatively and postoperatively until extraction site healed	82 (54.9%)	390	1/82 (1.2%)	1/390 (0.3%)
Aggarwal et al. (2017) [28]	Pentoxifylline 400 mg BID and Vitamin E 1000 IU daily, 1 month preoperatively and postoperatively until extraction site healed	110 (56.3%)	450	2/110 (1.8%)	Not reported
Samani et al. (2022) [29]	Pentoxifylline 400 mg BID and Vitamin E 1000 IU daily, 1 week preoperatively and postoperatively for 3 months	167 (Not reported) 148 *	879 775 *	6/167 (3.6%) 5/148 (3.4%) *	10/879 (1.1%) 8/775 (1.0%) *
Lombardi et al. (2022) [30]	Pentoxifylline 400 mg BID and Vitamin E 800 IU daily, 1 week preoperatively and postoperatively for 8 weeks	28 (58.7%)	152	3/28 (10.7%)	Not reported
Patel et al. (2023) [32] ^#^	Liquid pentoxifylline 200 mg QID and liquid vitamin E 1 g (10 mL) daily, 1 week preoperatively and postoperatively for 3 months	45 (71.1%)	Not reported	2/45 (4.4%)	Not reported
Current	Pentoxifylline 400 mg BID and Vitamin E 400 IU BID, oral tablets, 2 weeks preoperatively and postoperatively for 6 weeks	4 (100%)	8	0/4 (0%)	0/8 (0%)

* Patients with complete medication regimen compliance, ^#^ The majority of patients (38/45) had only dental extractions carried out (one patient had both extractions and implant placed and the remaining six patients had implants placed).

## Data Availability

Data are contained within the article.
